# Supplementary material for: Content Determination and Chemical Clustering Analysis of Tanshinone and Salvianolic Acid in Salvia spp
Source: Metabolites. 2024 Aug 8;14(8):441. doi: 10.3390/metabo14080441 (PMC11356371; doi:10.3390/metabo14080441)

Fig.S1 The content of phenolic acids in the roots of *Salvia spp.*

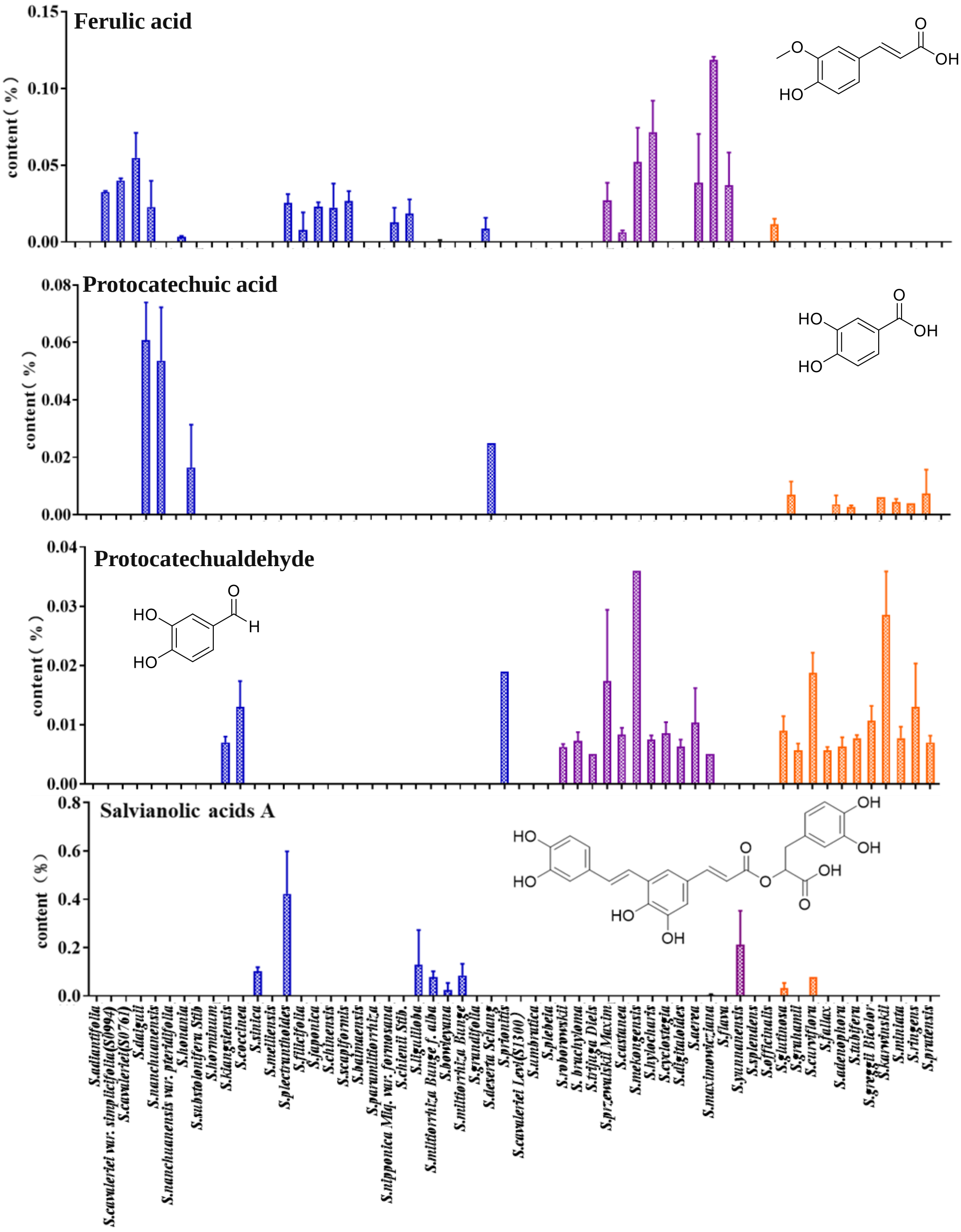

Fig.S2 The content of phenolic acids of leaves in *Salvia spp.*

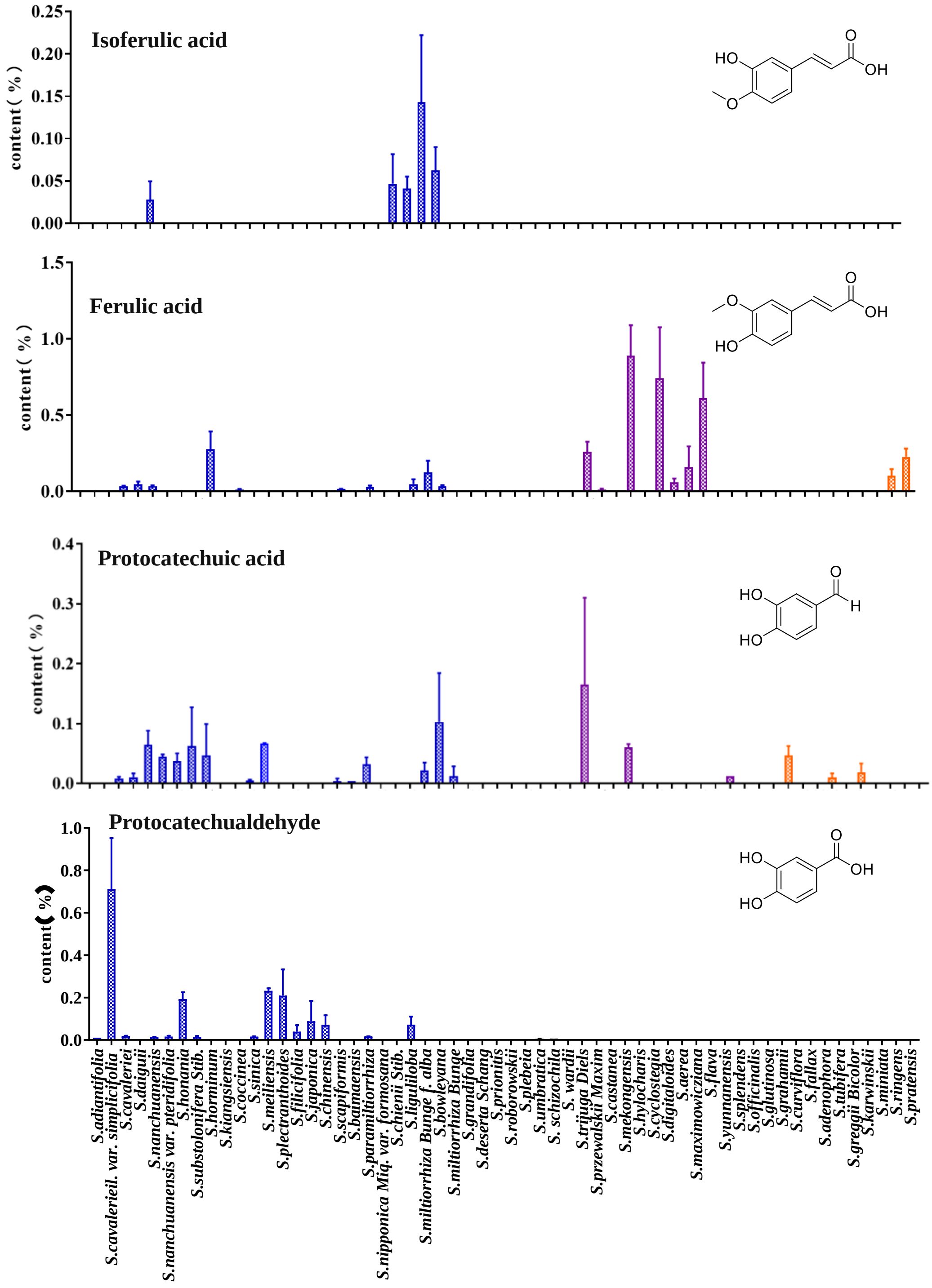

Supplement: Supplementary file 1 [file metabolites-14-00441-s001.zip › metabolites-3111043-supplementary.pdf]
